# Editorial: Innovative therapeutic approaches for complex cancers: exploring new strategies

**DOI:** 10.3389/fimmu.2026.1965922

**Published:** 2026-09-15

**Authors:** Vinay Kumar, Suman Asalla, Chaitenya Verma, Kavita Arora

**Affiliations:** 1 College of Medicine, Pennsylvania State University Hershey Medical Center, Hershey, PA, United States; 2 Department of Neurology, The Ohio State University Wexner Medical Center, Columbus, OH, United States; 3 Pelotonia Institute for Immuno-Oncology, James Comprehensive Cancer Center, The Ohio State University, Columbus, OH, United States; 4 Department of Biotechnology, School of Bio-Science and Technology, Sharda University Greater Noida, Delhi, Uttar Pradesh, India; 5 School of Computational & Integrative Sciences (SCIS) and Advanced Instrumentation Research Facility (AIRF), Jawaharlal Nehru University, New Delhi, India

**Keywords:** cancer, combination therapy, immunotherapy, theragnostic, therapy, tumor

## Introduction

Cancer remains a leading cause of death, with more than 20 million (20,636,441) cases reported worldwide each year. According to the Global Cancer Observatory (1), 50.7% of cancer cases occur in Asia). The cancer mortality rate is also the highest in Asia, at 56.7%, compared to the other continents, which vary between 0.79 and 20%. Lung cancer cases are the most prevalent among men, with breast cancer being the most prevalent among women, followed by colorectal, liver, and stomach cancers. Prostate and thyroid cancers are exceptions, being the third-highest and gender-specific for men and women, respectively. Rigorous diagnostic and therapeutic interventions or efforts are being made worldwide to address the growing need to treat cancer more effectively (2). Delayed diagnosis and ineffective therapeutic methods, especially for advanced-stage cancer, remain the primary cause of cancer-related mortality. Tumor surgical resection, chemotherapy, and radiotherapy remain an integral part of conventional cancer therapy to date. Various next-generation therapeutic methods are being introduced thanks to the rigorous research in this area; contemporary cancer-based therapies can largely be categorized as follows: (1) cell- and immuno-based therapies (e.g., CAR-T cell therapy, vaccinations, and immune checkpoint therapies) (3–5); (2) precision and molecular-targeted therapies (e.g., drugs, monoclonal antibodies, and small-molecular inhibitors) (6–8); (3) conjugate-based molecular assemblies for advanced delivery systems (e.g., smart biomolecular assemblies made of nanoparticles, micelles, antibodies, drugs, ligands, peptides, or aptamers) (3, 9); and (4) therapies that alter the tumor microenvironment with adaptive resistance (e.g., oncolytic viruses, peptidases, nanomaterials, drugs, and their combinations) (10, 11).

This Research Topic is an excellent collection of recent research that sheds light on some of the encouraging findings and approaches to various cancers, including glioblastoma, leiomyosarcoma, cold tumors, non-small cell lung cancer (NSCLC), pancreatic cancer, gastric cancer, bladder cancer, liver cancer including hepatocellular carcinoma, and urothelial carcinoma. This Research Topic includes three review articles and 14 research studies.

## Cold tumors

Cold tumors are known to be challenging because they are poorly recognized and infiltrated by the immune system and are characterized by low T-cell abundance. Han and team (Soko et al.) provided an overview of a review related to cold tumors and associated challenges towards effective immunotherapy. This work elaborated on the immune response or immunogenic cell death generated through oncolytic viruses (OVs), which work via inducing trafficking and infiltration of CD8^+^ T-cells into tumors. These cold tumors aberrantly express matrix proteins through cancer-associated fibroblasts (CAFs) and have an extracellular matrix (ECM) that provides extraordinary mechanical properties or desmoplasia and increased intratumoral interstitial fluid pressure (IFP). This creates physical barriers that decrease the penetration and dissemination of OVs within tumors. In this context, this review provides a comprehensive overview on the use of bio-chemical and physico-chemical strategies to degrade/inhibit/modulate tumor ECM i.e. intratumoral penetration. Biochemically, this tumor ECM, which is generally comprised of collagen, hyaluronic acid, and chondroitin sulfate proteoglycans (CSPG), can be induced for an immune response using matrix metalloprotease (MMP) and/or OV variants, pharmacological antifibrotic drugs (such as losartan or Halofuginone), or engineered OVs expressing DCN (Decorin with significant ECM-organizing capabilities). Physico-chemical strategies for ECM disruption utilizes magnetic (use of magnetic nanoparticles), sound, and light energies (photo dynamic therapy using light-sensitive drugs or nanoparticles) to physically or chemically disturb the tumor’s ECM. Furthermore, it was shown that the use of oncolytic adenoviruses or OVs can remodel tumor ECMs i.e., the dense tumor microenvironment, by lowering interstitial fluid pressure and reducing matrix stiffness to shift the tumor immune microenvironment (TIME) via tumor cell lysis or by overriding ECM-inflicted barriers on T-cell push in and intrusion into tumors. This review also summarizes 12 ongoing clinical trial studies, which are at phase I/II, and their associated challenges while elaborating on the high chances of translation of these therapies to clinical practice.

## Cervical cancer

Badou and his coworkers (Kouhen et al.) have reviewed existing therapies for cervical cancer, which has high recurrence and metastasis rates despite the availability of advanced chemoradiation therapies. It was described that immune checkpoint inhibitors can remarkably improve treatment by combining immunotherapy with standard chemoradiation (such as Cisplatin). Immune checkpoint inhibitors, tumor-invading lymphocytes, genetically modified T-cells for HPV-associated proteins, and antibody-drug conjugates offer immunogenic potential. This work also summarizes various Phase II and III clinical trials of immunotherapy, both as a monotherapy and in combination with chemoradiotherapy. It also highlights the role of biomarkers, including microsatellite instability (MSI) and tumor mutational burden (TMB), in guiding personalized treatment.

In the context of predictive biomarkers for cervical cancer, various factors have been identified so far. These included PD-L1 expression in tumor cells, tumor-invading lymphocytes, tumor mutational burden (TMB), microsatellite instability (MSI) and/or mismatch repair deficiency (MMR), tumor-draining lymph nodes (TDLNs), and the use of artificial intelligence (AI)-based screening systems (such as YOLOv3 for object detection, Xception and DenseNet-50 for target classification, U-net for nucleus segmentation, and XGBoost model for final slide-level), which offer opportunities to improve imaging, diagnostics, and the forecasting of clinical responses. Conclusively, these authors recommended the use of a synergistic combination of various therapeutic opportunities and agents to refine the clinical method and achieve maximum benefits while modulating various biochemical pathways and mitigating adverse effects to ensure immunotherapy and ensure better financial situations for a wider range of patients.

## Glioblastoma

Glioblastoma is a common and malignant brain tumor with a high rate of recurrence and mortality and an overall survival of < 2 years despite available standard therapies. Yuan and team (Zhou et al.) presented a comprehensive overview of treatment therapies combined with innovative diagnostic arenas for glioblastoma while elaborating on the associated challenges. They comprehensively described the major immunotherapeutic approaches for glioblastoma, including immune checkpoint inhibitors (such as PD-1/PD-L1 inhibitors, nivolumab, pembrolizumab, and cemiplimab), cancer vaccines (such as (1) cellular vaccines e.g., dendritic cell vaccines; (2) protein/synthetic peptide vaccines e.g., CDX-110 targeting EGFRvIII mutation, and IMA950 targeting multiple tumor specific antigens; (3) HSPPC-96, which is a heat shock protein-based vaccine targeting CD91; (4) the survivin-targeting vaccine-SurVaxM for glioma stem cells; (5) nucleic acid vaccines; and (6) viral vector vaccines), and chimeric antigen receptor T-cell (such as IL13Rα2, 308 EGFRvIII, and HER2) and oncolytic viral therapy (such as oncolytic herpes simplex virus, oncolytic adenovirus, poliovirus, retrovirus, Newcastle disease virus (NDV)-based vectors, and more). These therapies lack total success due to the intracerebral lymphatic system, challenges imposed by the blood-brain barrier, the highly immunosuppressive tumor microenvironment, hypoxia, heterogeneity, drug delivery obstacles, and low tumor mutation and its intrinsic features. Another major challenge is the logistical and financial burden of insurance. The use of nano particles may help solve the various challenges described above.

Immunotherapy based on chimeric antigen receptor (CAR) T-cells (12, 13) expressing human IL-15 and CCL19 was used by Chen et al. to improve therapeutic efficacy in human glioblastoma using an orthotopic mouse xenograft model. CAR T-cell therapy has been reportedly found to be effective in hematological malignancies only. Engineered CAR T-cells (15 × 19 CAR T-cells) helped overcome survival challenges in the tumor microenvironment, especially in solid tumors. These CAR T-cells exhibit excellent proliferation, chemotaxis, and phenotypic properties and have better control over tumors than conventional counterparts. The collective impact of IL15 and CCL19 has been shown to warrant the enhanced impact of this immunotherapy. As claimed, IL-15 facilitated the genesis, maintenance, and reactivation of naive T cells, effector T-cells, and memory T-cells and chemotactic factor CCL19 (C-C motif chemokine ligand 19) by facilitating the selection of T-cells and dendritic cells for better migration and infiltration.

Weighted gene co-expression network analysis (WGCNA) was implemented to identify 86 gene modules for Glioblastoma (GBM). This method offers aggressive progression, recurrence, and poor clinical prognosis. Wang and coworkers (Soko et al.) used clinical immunotherapy datasets to identify candidate genes associated with GBM and their response to immunotherapy. This process involved the use of multi-omics analyses across glioma, pan-cancer datasets, immune infiltration characteristics, and immunotherapy outcomes. Subsequently, through the machine learning pipeline (by employing four feature selection algorithms), 10 key regulators of anti-tumor immunity were identified and validated *in vitro* through high-throughput sequencing. It was revealed that PCLB4 genes play pivotal roles in cancer biology and have a role in tumor suppression and on the calcium signaling pathway. Candidate compounds were screened using molecular docking and CCK8 assays. Hence, this integrative approach paved the way for rational drug development by reducing unnecessary experimental screening and medical costs. This may also lead towards improving therapeutic outcomes and prognosis.

## Leiomyosarcoma

Li and colleagues (14) reported a palliative treatment methodology combining high-intensity focused ultrasound (HIFU) with first-line chemotherapeutic intervention (eribulin and sintilimab) to treat a case of aggressive leiomyosarcoma (LMS), a malignancy known for recurrence and metastasis. A successful demonstration suggested potential benefits through the partial response received in a 49-year-old patient with 7.2 months showing progression-free survival and 57-month overall survival compared to the therapeutic response for standalone systemic therapy. It was predicted that coagulative necrosis might have played a cytoreductive role by rapidly decreasing local tumor burden. Although this method warrants more investigation to attest for the multimodal approach for many more patients, these results appear to be promising.

## Pancreatic adenocarcinoma

Owing to intratumoral and interpatient heterogeneity, pancreatic adenocarcinoma (PDAC) is a formidable challenge for effective drug treatment. Yang and his group (Wang et al.) suggested the use of patient-derived organoids (PDOs) and xenografts (PDXs) and selecting synergistic combinations of drugs screened through sequencing somatic mutations of 425 oncogenes. Use of personalized therapy and synergistic combination of drugs such as MEK inhibitors (Trametinib) with mTOR (AZD8055) or CDK (Flavopiridol) inhibitors when tested on an established biobank of 27 tumor PDOs and 10 tested PDXs derived from 66 patient samples showed significant tumor suppression and translational utility of this treatment (both *in vivo* and *in vitro*) towards building a framework for customized clinical decision-making.

## Lung cancer

Lung Cancer is known to have the highest reported incidences in men worldwide, while NSCLC has the highest mortality rate. Ding and Zhang along with their research team (15) suggested detailed insights into the ROS1 rearrangement acquired for drug resistance to epidermal growth factor receptor-tyrosine kinase inhibitors (EGFR-TKIs) using next generation sequencing (NGS). They report that EGFR-TKI resistance is caused by the EZR exon 10–ROS1 exon 34 rearrangement as demonstrated in a case where the use of crizotinib and osimertinib provided favorable outcomes that overcame the acquired resistance. In fact, Crizotinib was reportedly cited to give a favorable response in an extensively reviewed small group.

## Gastric cancer

Xiong and colleagues (16) described a comparative study of 375 gastric cancer patients treated with neoadjuvant chemotherapy in the presence (NACI, 168 patients) and absence (NAC, 207 patients) of immunotherapeutic intervention (PD-1 inhibitors). The presence of PD-1 resulted in a significant decline in tumor (ypT0) burden without increasing the perioperative risk. The cytotoxic effects of chemotherapy can be augmented by combining it with immunotherapy, which works by activating T-cell-mediated immune responses. However, the prior safety assessment, pathological findings, and long-term prognosis of laparoscopic gastrectomy following therapy to both subgroups resulted in noticeable short-term therapeutic advantages, which had no impact on disease-free survival and overall survival for up to 3 years between the two groups.

## Liver cancer or hepatocarcinoma

Liver cancer has the third highest mortality amongst all cancers; Gao and Lan’s team (17) represented successful systematic combinatorial immunotherapeutic intervention in a rare case of 69-year-old patient suffering from synchronous double primary hepatocellular carcinoma and intrahepatic cholangiocarcinoma (sdpHCC-ICC) through the use of nivolumab plus, ipilimumab, and bevacizumab, achieving complete recovery of pulmonary lesions and partial response in hepatic lesions. Even though the sdpHCC-ICC was beyond surgical removal with pulmonary metastases, the use of combination immunotherapy with drugs helped achieve significant response for impacting metastasis and lesions. Subsequently, favorable long-term outcomes were obtained through subsequent surgical resection of the remaining tumor followed by postoperative adjuvant therapy.

## Urothelial carcinoma with acinar adenocarcinoma

A rare case of primary high-grade urothelial carcinoma of the prostate combined with acinar adenocarcinoma was subject to a new diagnostic and treatment strategy combining surgery with targeted immunotherapy regime. It was performed by an innovative port-free single-site robot-assisted radical prostatectomy (pf-ssRARP) followed by immuno-histochemical analysis (CK7/CK20/GATA3 positive, prostate-specific antigen (PSA) negative) and subsequent immunotherapy. The dual treatment protocol combined endocrine therapy (leuprorelin + rezvilutamide) for acinar adenocarcinoma with immunotherapy based on PD-1 inhibitor (disitamab vedotin + toripalimab) for urothelial components. The 61-year-old male patient showed no evidence of recurrence or metastasis during the 1-year follow-up after treatment.

## Bladder cancer

Urinary bladder malignancy is the fourth most common cancer affecting males worldwide, accounting for 6% of new cancer cases and 4% of cancer deaths. Zhou and his associates (Hu et al.) reported that the prognostic significance of platelet-to-lymphocyte ratio (PLR) in non-muscle invasive urinary bladder cancer (NMIBC) remains controversial. They systematically evaluated the prognostic value of PLR in NMIBC and suggested that it can be used as a prognostic biomarker and may serve as an independent predictor for relapse-free survival for NMIBC. It provides a cost-effective biomarker which is promising in risk stratification and treatment planning.

Metabolism-based biomarkers are known as important candidates for developing new strategies of therapeutic intervention and overcoming metastasis and chemotherapeutic resistance. The combination of consensus clustering and t-distributed stochastic neighbor embedding (tSNE) was very well demonstrated by He and his collaborators (Zhang et al.) in bladder cancer patients. They identified that lncRNA (LINC01094) mediated gene regulation using multiomics analysis for lactate metabolism-based modulation of tumor microenvironment (TME). This lncRNA gene caused metastasis and chemotherapy resistance by stabilizing the VIM (vimentin, a key mesenchymal biomarker) protein for tumor progression and inhibiting its ubiquitination. This gene was identified as a potential target for gene therapeutic intervention. It was suggested that bladder cancer, which is prone to metastasis and responds poorly to chemo/immuno-therapy, now has the possibility of better therapeutic targeting using LINC01094 with antisense oligonucleotides to decrease cell metastasis and enhance the effect of chemotherapy.

Chen and his team (Cai et al.) presented the development of a novel predictive and prognostic model for bladder cancer. The SCAMP2 gene was identified as a key regulator from the cisplatin sensitivity-related genes (CSRGs) family especially to cater to resistance developed to platinum-based chemotherapy drugs. Further, the associated drug-related response/resistance (related to NOTCH signaling pathway) was then cross verified using *in vitro* and *in vivo* experiments that suggested that drug resistance can be overcome through personalized treatment strategies and potential therapeutic targets.

Zhuang and his colleagues (Cai et al.), delineated a gender-based disbalance in bladder cancer prognosis and reported the critical role of the XIST/miR-15a-5p/MN1/FZD2 signaling axis identified through comprehensive bioinformatics analysis and experimental functional/molecular analysis carried out in cancer cell lines and in *in vivo* validation in zebra fish. The XIST/miR-15a-5p/MN1/FZD2 signaling axis is involved in the long non-coding RNA X-inactive specific transcript (XIST) expressed on X chromosome coding for small non-coding regulatory RNA molecule ‘miR-15a-5p’ while coexisting with the oncogene MN-1 (menin 1 or meningioma 1), which upregulates the expression of the downstream gene FZD2. There is a strong correlation between XIST and poor prognosis among females, thereby warranting its potential as a novel therapeutic target.

Muscle-invasive bladder cancer (MIBC), an aggressive form of bladder cancer, requires combinatorial treatment for better efficacy. This concern was addressed by Zhang et al., 2025, who studied 38 patients (17 + 21) to understand and compare the efficacy and safety of Disitamab Vedotin (ADC) in combination with PD-1 (programmed cell death protein-1) inhibitors with the conventional gemcitabine and cisplatin (GC) regimen when used as a neoadjuvant therapy in patients subject to surgical resection. Interestingly, pathological complete response rates (PCRR) reported a significantly higher response for ADC + PD-1, underscoring the potential of antibody-drug conjugates within the field of cancer immunotherapy. This may address critical gaps in current treatment methodologies and suggest novel pathways for future clinical applications.

Liu and his colleagues (Han et al.) identified a set of five crucial long non-coding RNAs (lncRNAs) related to disulfidptosis for bladder cancer prognosis and treatment, suggesting sensitivity to drugs Sorafenib and Oxaliplatin. Genes associated with the PI3K-Akt signaling pathway showed higher expression, resulting in extracellular matrix organization and immune escape mechanism.

Against the backdrop of this wide range of therapeutic research investigations on various cancers and subsequent interesting developments, it is apparent that the use of combinatorial therapies and bioinformatic-based tools will open up insights into various biochemical pathways that might help uncover new regimes for achieving improved and customized cancer drugs and therapies.

## Conclusions and future prospects

These research articles throw a spotlight on the importance of combinatorial therapy (such as drugs, radiotherapy, surgical resection, immunotherapy, CAR T-cell therapy, patient-derived organoids, xenografts, and many more) and the incorporation of new, nano-molecular assemblies (nanomaterials + drugs), meta-analysis, multi-omics based analysis, transcriptome analysis, and machine learning-based strategies to streamline cumbersome experimental outcomes. Additionally, the use of nanotechnology and combinatorial or chimeric-molecular assemblies have further warranted their use in specific cancers alongside theranostic applications i.e., offering therapy and diagnosis in one go. These therapeutic approaches have opened new avenues for leveraging bioinformatics tools in combination with artificial intelligence and machine learning for molecular analysis. These technologies facilitate the identification of target genes and cancer-specific molecular alterations and the prediction of potential therapeutic drugs, biomolecules, receptors, or biomarkers. Such advances may enable the development of personalized therapies to overcome treatment resistance and improve outcomes in advanced-stage cancers, ultimately leading to improved patient survival.
